# The health worker recruitment and deployment process in Kenya: an emergency hiring program

**DOI:** 10.1186/1478-4491-6-19

**Published:** 2008-09-16

**Authors:** Ummuro Adano

**Affiliations:** 1 Management Sciences for Health, Cambridge, MA, USA

## Abstract

Despite a pool of unemployed health staff available in Kenya, staffing levels at most facilities were only 50%, and maldistribution of staff left many people without access to antiretroviral therapy (ART). Because in the current system it takes one to two years to fill vacant positions, even when funding is available, an emergency approach was needed to fast-track the hiring and deployment process.

A stakeholder group was formed to bring together leaders from several sectors to design and implement a fast-track hiring and deployment model that would mobilize 830 additional health workers. This model used the private sector to recruit and deploy new health workers and manage the payroll and employment contracts, with an agreement from the government to transfer these staff to the government payroll after three years.

The recruitment process was shortened to less than three months. By providing job orientation and on-time pay checks, the program increased employee retention and satisfaction.

Most of the active roadblocks to changes in the health workforce policies and systems are 'human' and not technical, stemming from a lack of leadership, a problem-solving mindset and the alignment of stakeholders from several sectors.

It is essential to establish partnerships and foster commitment and collaboration to create needed change in human resource management (HRM).

Strengthening appointment on merit is one of the most powerful, yet simplest ways in which the health sector and governments that seek to tackle the challenges of corruption and poor governance can improve their image and efficiency.

The quality and integrity of the public health sector can be improved only through professionalizing HRM, reformulating and consolidating the currently fragmented HR functions, and bringing all the pieces together under the authority and influence of HR departments and units with expanded scopes. HR staff must be specialists with strategic HR functions and not generalists who are confined to playing a restricted and bureaucratic role.

## Introduction

In Kenya, a public-sector hiring freeze beginning in 1994 has resulted in a shrinking health workforce that limits the government's ability to respond to increased demand for health services. Although the government announced an ambitious program to expand access to HIV and AIDS, tuberculosis, malaria, and other health services, the lack of health workers meant that hospital beds and floors quickly filled up with patients who were ill or dying from AIDS-related illnesses. This crisis occurred even though Kenya has a substantial pool of qualified health professionals, especially nurses, who are unemployed and available on the local labour market and patients with AIDS-related diseases can usually be discharged once they are started on ART and have been stabilized. The initial phases of the Emergency Hiring Program, therefore, focused on the Nyanza, Western, and Coast provinces, where the need for AIDS treatment was the most severe, but the program later covered all the remaining provinces, including remote and hard-to-reach facilities with a less severe AIDS burden.

One of the major challenges to developing sustainable health systems in sub-Saharan Africa is lack of human resources. In Kenya, a shrinking public health workforce, staffing levels of 50% at most facilities, and maldistribution of existing staff contribute to the fact that thousands of people living with AIDS, especially in rural areas, do not have access to ART. These staff shortages resulted from migration, a long freeze on civil service employment, and a high rate of attrition due to the impact of AIDS and poor working conditions – a common scenario.

Provinces like Nyanza and Western bear the largest burden of the health worker shortage. At Nyando District Hospital, AIDS-related conditions afflict 99% of the adult patients. Each day over 100 new patients flock to the hospital, yet just one doctor, fifteen nurses, and four clinical officers are on hand to cover all shifts. The lean staff mean that patients often wait for long periods to get attention and quality of care suffers. Staff burnout is a problem. The irony is that there is a large pool of trained, unemployed health workers available in Kenya, but the process of recruitment, hiring, and deployment is outdated and bureaucratic, taking up to 18 months to complete.

The US Agency for International Development, in consultation with the Ministry of Health (MOH), approached the Capacity Project to address this challenge. A Management Sciences for Health (MSH) specialist seconded to the project led the process of developing an emergency hiring plan to expand HIV& AIDS services in Kenya's public health sector. Stakeholders such as the MOH, Directorate of Personnel Management in the Office of the President, Ministry of Education, and Ministry of Finance came together to consider options.

A business model with responsive and flexible procedures was adopted, and local Kenyan organizations with proven capability and experience were identified and contracted to develop and implement the plan.

## Discussion

### Description of the Emergency Hiring Plan

An agreement was reached with the stakeholder team to form an entity to hire qualified health professionals on short-term contracts to staff facilities. The core business functions of this new entity are:

▪ staff attraction

▪ screening and selection

▪ recruitment

▪ training

▪ deployment

▪ payroll and benefits management

▪ retention.

Deloitte & Touche, Kenya, was selected to carry out most of these business functions, and the African Medical and Research Foundation, Kenya Medical Training College, and Kenya Institute of Administration were selected to work together to ensure that the newly hired providers have the necessary knowledge and skills to provide HIV and AIDS services. All training is consistent with national standards and guidelines already in use.

### Recruitment and deployment

It was estimated that approximately 5000 nurses, 1000 clinical officers, 1200 laboratory staff, and 160 pharmacists were unemployed and potentially available for hire. The recruitment criteria aimed not to pull workers out of the public health care system, cause resentment among existing workers through the introduction of inequitable compensation plans, or draw from the private sector or faith-based organizations and reduce their effectiveness. The recruitment approach focused on the same geographic areas where staff were needed, in the expectation that people would be less likely to want to transfer if they worked close to home.

### Compensation package

The compensation package was developed in line with MOH standards and terms and conditions of service. These workers are accountable to the MOH reporting and supervision system, and an agreement was negotiated that they will all be absorbed into the government system after three years.

To date, 830 health staff have been hired, trained, and deployed in 219 public health facilities in approximately six months through the Emergency Hiring Program. The new hires are given three-year contracts and then will become permanent MOH staff. While the program has already made a difference – for example, a large hospital near the border with Sudan was scheduled to be closed, but 15 nurses hired by the program are keeping it open and providing services for this remote region – the Emergency Health Program is a pilot project that must be carefully evaluated.

One of the key lessons that this pilot in Kenya brought to the forefront was the inevitable tension between effecting long-lasting, fundamental change and rapid change to respond to an emergency – a tension common in most countries in sub-Saharan Africa. Stopgap measures must go hand-in-hand with long-term systemic interventions.

The project strategy remains two-pronged. Initially the Emergency Hiring Program was used to put the necessary trained staff in place quickly, but gradually the focus is shifting towards making fundamental changes in the HRM system (planning, workforce data, safe working conditions, public service reform, and pre-service and in-service education).

## Conclusion

There are several success factors and next steps to take to move this emergency program forward towards more sustainable change:

▪ transparent and thorough monitoring and evaluation of the Emergency Hiring Program;

▪ documentation of best practices;

▪ commitment of the stakeholder group to move toward HR reform;

▪ continued effort to build leadership and management capacity at all levels;

▪ professionalizing HR departments and units and ensuring that HR staff have input into strategic decisions and HR innovations that will strengthen the performance of the health system;

▪ development and support of HR leaders who have the capacity to motivate, communicate, and lead change in order to create commitment and a shared vision.

Leadership is critical because even a successful hiring program cannot be scaled up or combined with national HR reform efforts without health staff at all levels who can lead and manage the reform and translate it into positive results on the ground.

## Competing interests

The authors declare that they have no competing interests.

